# Symptoms of an Intrauterine Hematoma Associated with Pregnancy Complications: A Systematic Review

**DOI:** 10.1371/journal.pone.0111676

**Published:** 2014-11-04

**Authors:** Lan Xiang, Zhaolian Wei, Yunxia Cao

**Affiliations:** Reproductive Medicine Center, the First Affiliated Hospital of Anhui Medical University, Hefei, China; Central South University, China

## Abstract

**Objective:**

To evaluate the predictive value of the symptoms of an intrauterine hematoma (IUH) for adverse pregnancy outcomes.

**Methods:**

A literature review was performed with the search terms, including intrauterine/subchorionic/retroplacental/subplacental hematoma/hemorrhage/bleeding/collection/fluid, covering the period from January, 1981 to January, 2014. We just focused on the pregnancy outcomes associated with different symptoms of an IUH.

**Results:**

It is generally agreed that a retroplacental, posterior or subchorionic in the fundus of uterus, and/or persistent IUH is associated with adverse outcomes in the ongoing pregnancy. However, the prognosis value of both volume and gestational age at diagnosis of IUH still remains controversial. Some researchers argue that a large IUH is associated with an increased risk of adverse events during pregnancy while others refuted. It is believed by some that the earlier an IUH was detected, the higher the risk for adverse outcomes would be, while no or weak association were reported by other studies. The prognostic value of the simultaneous presence of vaginal bleeding on pregnancy outcome is also controversial.

**Conclusions:**

Both the position relative to the placenta or uterus and duration of IUH have strong predictive value on the prognosis in the ongoing pregnancy. However, the prognostic values of the IUH volume, gestational age at diagnosis and the simultaneous presence of vaginal bleeding remain controversial up to now. Moreover, most of previous reports are small, uncontrolled studies with incomplete information. Prospective, large sample, cohorts studies which take all detailed symptoms of an IUH into consideration are needed when we evaluate its clinical significance in the prognosis of pregnancy.

## Introduction

Intrauterine hematoma (IUH) is a common phenomenon on routine obstetric ultrasonography, especially in the first trimester of gestation. The reported incidence of IUH ranged from 0.46% to 39.5% [1], [2], depending on the populations studied, definition and gestational age at diagnosis. The clinical significance of IUH has always been controversial since it was first described in 1981 [3]. Some studies hypothesized that the presence of IUH is strongly associated with adverse events during pregnancy, including gestational hypertension, pre-eclampsia, placenta abruption, preterm delivery (PTD), small for gestational age (SGA) and low 5-min Apgar score [4]. However, others found no association between the IUH and those adverse perinatal outcomes [2], [5].

A number of studies have sought to identify the adverse outcomes at the presence of IUH in pregnancies, but only a few systematic reviews have been made. An old review in 1993 reported the incidence and the small SCH was common in the first trimester and posed no additional risk to the ongoing pregnancy [6]. A recent meta-analysis demonstrated that the SCH was associated with an increased risk of early and late pregnancy loss, abruption, and preterm premature rupture of membranes, covering only seven cohort or case-control studies [7]. Both of the two reviews simply described the association of SCH between with pregnancy complications. However, multiple factors, including the volume and location of IUH, diagnosed gestational age, duration and the simultaneous presence or absence of vaginal bleeding, may play important roles in the prognosis for pregnancy outcomes [1], [3], [8], [9]. Therefore, the aim of this article was to systematically review the literatures on the relevant symptoms of a hematoma associated with pregnancy complications.

## Materials and Methods

### Search strategy

We performed a systematic literature search using the computerized databases Pub Med and EMBASE, covering the period between January 1981 (since the first report on IUH was published in that year) and January 2014. The searches were restricted to the English language. In addition, the citation lists were independently reviewed to identify for cross-references. For the purpose of this update, a comprehensive search was conducted abiding by the following search strategy.

**Figure 1:**
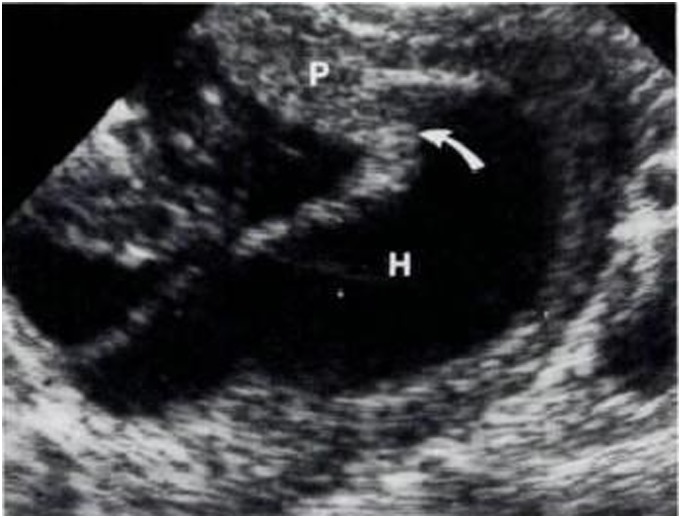
A resolving subchorionic hematoma (H), detected at 13 menstrual weeks, extending beneath the margin (arrow) of the placenta (P) [8].

**Figure 2:**
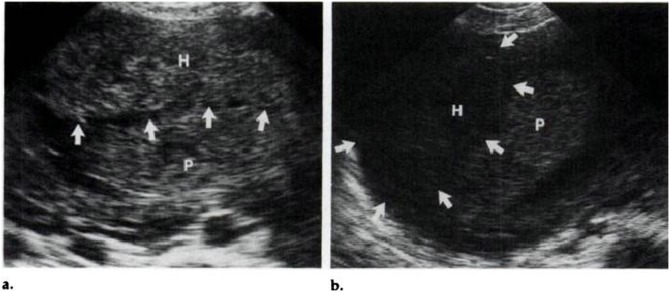
A large retroplacental hematoma (H) detected at 25 menstrual weeks, detaching more than 50%of the placenta (P). Retroplacental venous complex (arrows) separated the hematoma and placenta (a); 1 week later, a resolving hematoma (H) contained (arrows) posterior to the placenta (P) (b) [8].

**Figure 3:**
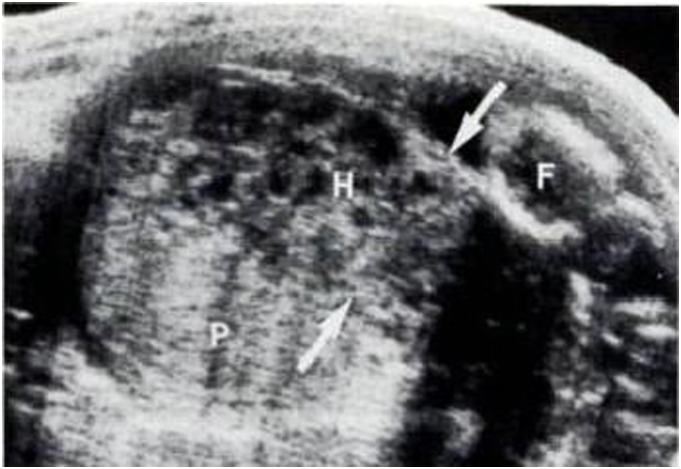
A preplacental hematoma (H), detected at 18 menstrual weeks, located between the placenta (P) and the fetus (F) [8].

IUH was divided into three types according to their locations [8], [10]: subchorionic (SCH, between the myometrium and the placental membranes and/or at the margin of the placenta, 81%, Figure 1), retroplacental (between the placenta and the myometrium, 16%, Figure 2), and preplacental (between the placenta and the amniotic fluid/placental membranes, 4%, namely called subamniotic hematoma later [11], Figure 3) Because of the low incidence of preplacental type, and most discussions focused on the first two types in published literatures, we reviewed subchorionic and retroplacental hematomas in this article. In addition, massive subchorionic hematoma/thrombohematoma (the so-called ‘Breus’ mole’, a hematoma placed beneath the chorionic plate separating it from the underlying intervillous space and must be more than 1 cm thick), different from those two hematomas [11]–[13], was also excluded in our review. Search term combination for bibliographic databases was text words, including the following variations of search terms combing with pregnancy outcome: intrauterine/subchorionic/retroplacental/subplacental hematoma/hemorrhage/bleeding/collection/fluid. In view of the heterogeneity of the data, we did not apply a formal meta-analysis in this review.

### Study selection

**Figure 4:**
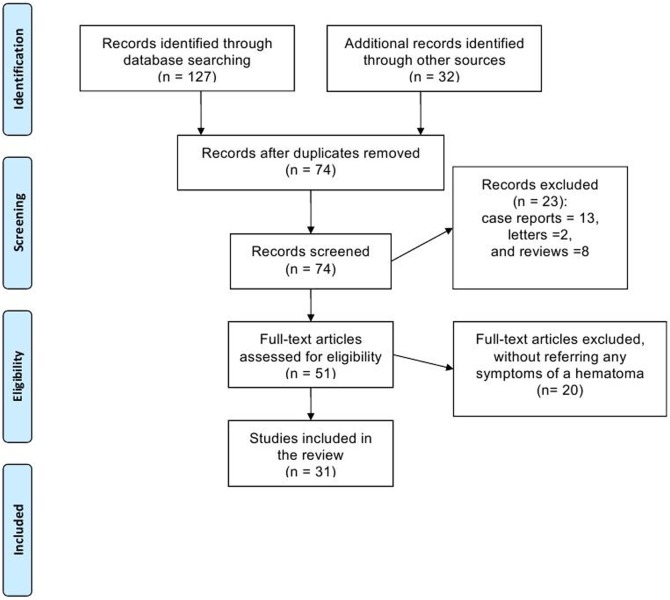
Flow diagram of studies in the review.

We identified all prospective and retrospective studies on IUH regardless of whether or not a control group was made. Studies described none of the following: volume, location or position, duration, gestational age at diagnosis of IUH, or vaginal bleeding, were excluded. Case reports, letters, and reviews were excluded too. Details for the flow diagram of studies in this review were presented in Figure 4.

In this review, we just focused on the pregnancy outcomes associated with different symptoms of an IUH. To make an overview, the information of all studies, including year of publication, details on the symptoms of an IUH, and relevant pregnancy outcomes, was listed in Table 1.

**Table 1:** Symptoms of An Intrauterine Hematoma Associated with Pregnancy Complications.

Study	StudyDesign	StudyParticipants	ExclusionCriteria	Definition of IUH	OutcomesEvaluated(Definitions)	Definition ofAbsoluteVolume	Criteria forAbsoluteVolumeClassification	Criteria for RelativeVolume Classification(in Related toGestationalSac Size)	SiteRelatedto thePlacenta	LocationRelated tothe Uterus	CriteriaforGestational AgeClassification	Duration of AHematoma	InvolvingVaginalBleeding or Not
Mantoni et al. (1981)	prospective	12 pregnancieswith a live fetusand hematoma inthe threatenedabortion population(11–20 weeks)	––	IUH: an echo-free area between the uterine wall and the membranes	abortion and PTD (not defined)	product of longitudinal, transverse, and antero-posterior diameters	>50 ml,<35 ml, <5 ml	––	elevate a part of or reach close to the plcenta border	on the posterior wall, close to, or covering, the internal os	>16 weeks, 12–16 weeks	disappearing by the 20th week (<35 ml) or persistance (>50 ml)	yes
Goldstein et al. (1983)	prospective	56 pregnanciesbetween 9 and 16weeks of gestationwith symptoms oflower abdominalcrampy pain andvaginal bleeding	≤8 weeks	subchorionic bleeding: cresentic fluid collection between the gestational sac and the uterine wall	term delivery and fetal death (not defined)	––	––	––	begin near the edge of placenta, extending to various distances from the placenta	––	9–10 weeks,11–12 weeks, 13–14 weeks	resolution over 4–7 weeks or persistance until fetal death	yes
Ylöstalo et al. (1984)	prospective	26 patients withuterine bleedingat 12–33 weeksof pregnancy	––	IUH (not defined)	placental abruption (not defined)	––	––	––	behind the placenta, marginal to the placenta, or remote from the placenta	––	––	––	yes
Jouppila (1985)	prospective	33 singlepregnancies with both uterinebleeding andhematoma (8–17weeks)	Cases with thickwalled secondary sacs separate from the main amniotic cavity	IUH: a crescent-shaped echo-free area outlining the intact gestational sac or an echo-free area between the uterine wall and the fetal membranes from the 13th week onward	abortion (not defined), PTD (<37 weeks)	mean of its longitudinal, transverse, and anterioposterior diameters	≥4 cm, <4 cm	––	arising from the placental margin	––	––	totally disappearing by the 24th week in the successful pregnancies	yes
Mantoni (1985)	prospective	260 consecutivepregnancies withvaginal bleeding	16 patients with bleeding originating from a cervical plop or erosion and unpregnancies	IUH: an echo-free area between the uterine wall and the membranes	abortion (not defined)	––	––	––	––	––	––	disappearing by the 25th week	yes
Abu-Yousef et al. (1987)	prospective	21 pregnancieswith subchorionichemorrhage (8–19 weeks)	––	SCH: elevation of the chorionic membrane on the side of the uterus opposite the placenta	abortion (<20 weeks),PTD (<36 weeks)	half of the product of its longitudinal, transverse, and anterioposterior diameters	>45 ml, <30 ml	small(<20%), medium(20–40%), and large(>40%)	elevation of the placental margin or not	––	––	patients with an increase or no change in the size of the hematoma had unfavorable outcomes, whereas only 33% of patients with a decrease in the size of the hematoma had unfavorable outcomes	yes
Sauerbrei et al. (1986)	prospective cohort	study group: 30pregnancies withboth vaginalbleeding and subchorionichematoma (10–20 weeks) control group: 30 pregnancies without hematoma selected at random from the same time period	––	SCH: subchonionic fluid collection	PTD (<37 weeks), abortion, stillbirth (not defined)	product of longitudinal, transverse, and anterioposterior diameters	>60 ml, <60 ml	small(<40%), large(>40%)	detachment of the placental margin	––	––	diappearing or decreasing in 2–4 weeks, or persistance for 8–12 weeks	yes
Stabile et al. (1987)	prospective	624 pregnancies with vaginal bleeding	158 women without clinical or ultrasonic evidence of pregnancy	IUH: (not defined)	miscarriage (fetal parts were clearly demonstrated but no fetal heart action was present)	––	––	––	––	––	––	––	yes
Nyberg et al. (1987)	retrospective	69 consecutive pregnancies with placental abruption or placental hematoma	hemorrhages associated with placenta previa	SCH (located predominantly between the myometnium and placental membranes and/or at the margin of the placenta); retroplacental hemorrhage (located between the placenta and myometnium), preplacental hemorrhage (located between the placenta and placental membranes)	perinatal death, premature labor and/or premature delivery of a living infant between 20 and 36 menstrual weeks, small for gestational age (defined as a birth weight less than the 10th percentile predicted by menstrual age and sex), normal term delivery	product of longitudinal, transverse, and anterioposterior diameters multiplied by the constant 0.52	>60 ml, <60 ml	––	subchonionic, retroplacental, or preplacental hemorrhage.	––	<20 weeks, >20 weeks	––	yes
Mandruzzato et al. (1989)	prospective	62 pregnancies with both vaginal bleeding and IUH (6–17 weeks)	––	IUH: an anechoic area that has a falciform shape, and is usually observed behind or below the intact gestational sac	PTD (<36 weeks), abortion, IUGR (not defined)	product of longitudinal, transverse, and antero-posterior diameters	small (<15 ml), large(>15 ml)	––	––	––	––	––	yes
Børlum et al. (1989)	prospective cohort	380 patients with a living fetus (>8weeks)	less than 9 weeks, ongoing abortion, blighted ova, more than one gestation or no intrauterine gestation	IUH: an echo-poor or echo-free crescent-shaped collection between the choronic membrane and the myometrium	abortion and PTD (not defined)	half of the product of its longitudinal, transverse, and anterioposterior diameters	small (<10 ml), medium(10–30 ml), large(>30 ml)	––	––	––	the first or second trimester	––	yes
Bloch et al. (1989)	prospective	31 single pregnancies with both first-trimester bleeding and subchorionic hemorrhage	––	SCH: a crescent-shaped anechoic collection or hypoechoic fluid	abortion, premature labor, Apgar score(not defined)	––	––	––	near the edge of the placenta extending a few millimeters from the placental site	––	––	persistance up to the 12th week	yes
Pedersen et al. (1990)	prospective	23 pregnancies with a live fetus, presenting with both vaginal bleeding and a large hematoma (≥50 ml) (12–20 weeks)	––	IUH: an intrauterine echo-free area between the uterine wall and the membranes	PTD(<36 weeks), abortion, neonatal death (not defined)	half of the product of its longitudinal, transverse, and anterioposterior diameters	large(≥50 ml)	––	typically elevating the border of the placenta	locating posteriorly, or laterally and anteriorly when the placenta completely occupies the posterior uterine wall	––	––	yes
Pedersen et al. (1990)	prospective	342 pregnancies who had vaginal bleeding with a live fetus (9–20 weeks)	patients electing to have an abortion and lost to follow-up and hematomas smaller than 2 ml were excluded	SCH: an echo-free or echo-poor intrauterine area outside the membranes	abortion (not defined) and PTD (≤day 252)	half of the product of its longitudinal, transverse, and anterioposterior diameters	––	––	––	––	9–11 weeks, 12–14 weeks, 15–20 weeks	disappearing by the 24th week	yes
Glavind et al. (1991)	retrospective	60 patients with a live fetus and an intrauterine hematoma (7–24 weeks)	one patient ending in a therapeutic abortion	IUH: an echo-free crescent shaped area between the membranes of the intact gestational sac and the uterine wall	abortion and PTD (not defined)	the largest diameter observed	––	––	subplacental or subchorionic localization	––	––	hematomas were present for a period of median 6 weeks(range O-22 weeks).	yes
Dickey et al. (1992)	retrospective	2899 normal pregnancies (5–8 weeks)	––	Subchorionic Fluid: sonolucent, crescent- or wedge-shaped areas between the uterine wall and chorion	embryonic death (a fetal heart rate was not detected on or after the 8th gestational week)	––	––	small(inferior or superior to the gestational sac or a thin line of fluid along the gestational sac wall), mpoderate(equal to 50% of geataional sac size at 5–8 weeks' gestation, or extend along the sac wall at 8–12 weeks'gestation), large(>50% of geataional sac size at 5–8 weeks' gestation, or multiple collections noted around the sac wall at 8–12 weeks' gestation)	––	––	––	––	yes
Rizzo et al. (1995)	prospective	38 pregnancies with bleeding (9–14 weeks): (1)singleton pregnancy; (2)certain last menstrual period; (3)live fetus; (4)presence of retroplacental hematoma; (5)successful recordings in all the vascular districts considered for the study; (6)exhaustive perinatal follow-up	––	Retroplacental Hematoma: an echo-free area separating the placenta from the uterine wall	abortion (not defined)	half of the product of its longitudinal, transverse, and anterioposterior diameters	––	––	––	––	––	––	yes
Ball et al. (1996)	case-control	24,291 obstetric patients	absence of fetal heart motion, fetal malformations, muhiple gestations, and patients who underwent elective terminations	SCH: a hypoechoic area between the chorion and the uterine wall.	abortion(<20 weeks), stillbirth (>20 weeks), and neonatal death(death in first 28 days of life)	––	––	small(≤5%), medium(5–25%), and large(≥25%)	––	––	––	––	yes
Bennett et al. (1996)	retrospective	516 pregnancies with only a live fetus (6–13 weeks), presenting with both vaginal bleeding and subchorionic hematoma	37 patients without follow-up information	SCH: an anechoic area that separated the chonion from the inner aspect of the uterus with a collection of fluid in the intrauterine cavity	abortion(not defined)	––	––	small(<1/3), medium(1/3–1/2), and large(≥2/3)	––	––	>8 weeks, ≤8 weeks	––	yes
Kurjak et al. (1996)	case-control	study group: 59 pregnancies with vaginal bleeding, closed cervix, and ultrasonic findings of a living embryo and subchorionic hematoma; control group: 135 pregnancies randomly selected and matching for maternal age, parity, and gestational age (6–14 weeks)	––	SCH: an echo poor or echo free crescent shaped collection between the chorionic membrane and myometrium	abortion, PTD (not defined)	half of the product of its longitudinal, transverse, and anterioposterior diameters	>20 ml, <20 ml	––	––	fundus-corpus, or supracervical, of the uterus	––	––	yes
Seki et al. (1998)	retrospective	22 pregnancies with persistent subchorionic hematoma with symptoms of vaginal bleeding or uterine contractions until delivery	patients whose clinical symptoms or subchorionic hematoma vanished later	SCH: an echo-free area located between the membranes and the uterine wall unassociated with a placenta	abortion,premature labor, premature rupture of membranes (not defined)	––	>30 ml	––	at the edge of the placenta	––	––	persistance until delivery	yes
Signore et al. (1998)	retrospective case-control	study group: 167 singleton pregnancies with vaginal bleeding (13–26 weeks); control group: 167 pregnancies obtained by selecting the next consecutive patient (singleton pregnancies and no history of second-trimester bleeding)	––	intrauterine clots	preterm delivery(<37 weeks), fetal death(at any gestational age), early neonatal death(0 to 7 days), neonatal intensive care unit admission, low umbilical artery blood PH(<7.20), fetal growth restriction, and cesarean delivery for fetal distress	––	––	––	––	––	––	––	yes
Tower and Regan (2001)	case-control	341 patients with viable pregnancies in a recurrent miscarriage population (≥6 weeks)	––	IUH: an crescent-shaped echo-free area between the uterine wall and the membranes	miscarriage, live birth, pre-eclampsia, PIH, IUGR, placenta praevia, abruption(not defined), PTD(<37 and <32 weeks)	––	––	––	––	––	––	resolved before the end of the first trimester in most cases	yes
Nagy et al. (2003)	prospective cohort	6675 pregnancies with a viable,singleton gestation (5–12 weeks)and delivery after 24 weeks’gestation.	patients with a nonviable fetus, multifetal pregnancy,or fetal abnormality	IUH: a crescent-shaped,sonolucent fluid collection behind the fetal membranes or the placenta	PIH, preeclampsia, placental abnormalities (placental abruption, cotyledon retention, and retained placenta requiring manual removal), meconium-stained amniotic fluid, fetal distress(persistent late decelerations or other heart rate patterns consistent with fetal hypoxia), preterm birth (<37 weeks), fetal growth restriction (a birth weight of less than the 10th percentile), and NICU admission	product of longitudinal,transverse,and anterioposterior diameters multiplied by the constant 0.52	––	small(<20%),medium(20–50%),and large(>50%)	subchorionic hematoma (between the chorion and the uterine wall), retroplacental hematoma (behind the placenta)	anterior, posterior, fundal, or cervical	––	––	yes
Sharma et al. (2003)	retrospective	129 single pregnancies with a subchorionic echolucency	Pregnancies with retroplacental collections	Subchorionic Echolucency: an echolucent area juxtaposed between the chorionic plate and placenta or chorion and decidua vera	Pregnancy loss (<24 weeks), PTD, Intrauterine growth restriction(IUGR) (defined as birth weight less than the 10% for gestational age using United States data)	maximum area or dimension	––	––	––	––	first, second, third trimester	––	yes
Ben-Haroushet al. (2003)	retrospective	230 women of threatened abortion with both a singleton living embryo or fetus and subchorionic hematoma (7–20 weeks)	––	SCH: a crescent-shaped echo-free area outlining the intact gestational sac in the first trimester, and an echo-free, usually elongated area between the uterine wall and the fetal membranes beyond 13 weeks gestation	abortion (not defined), PTD(≤37 weeks)	mean diameter of the transverse, sagittal and coronal planes	<4 cm, = 4 cm	––	subchorionic hematoma (between the chorion and the uterine wall, external to the chorion laeve), retroplacental hematoma (behind the placenta, external to the chorion frondosum), or both	––	10–12 weeks, 13–20 weeks	––	yes
Maso et al. (2005)	retrospective	182 pregnancies with a viable live fetus	patients who underwent elective abortion and/or invasive procedures and cases with multiple pregnancies, recurrent miscarriage(with a history of ≥2 consecutive first-trimester losses), uterine pathology(myomas), and malformations	IUH: an echo-free area between the uterine wall and the membranes	abortion(<20 weeks), fetal growth restriction (birth weight <10th percentile), PTD (<37 weeks), intensive care for threatened preterm delivery (need of admission and tocolytic therapy), placental abruption (a clinically relevant event determined by the managing physician) and fetal distress (abnormal fetal heart monitoring traces or fetal blood sampling suggestive of hypoxemia/acidemia)	product of longitudinal, transverse, and anterioposterior diameters multiplied by the constant 0.523	small (<1 ml), medium(1–10 ml), large(>10 ml)	––	––	––	<9 weeks, ≥9 weeks	––	yes
Leite et al. (2006)	prospective	30 pregnancies with single intrauterine live pregnancy and the detection of a very large hematoma (5–14 weeks)	patients with multiple pregnancies, nonviable or nonvisible embryos, and pathologic features, including fibroids, polyps, and uterine malformations, and those who underwent elective termination of pregnancy	IUH: a mostly crescent-shaped collection below the placenta or fetal membranes	abortion, PTD, or premature rupture of membranes (not defined)	––	––	small(<20%),medium(20–50%),and large(>50%)	subchorionic hematoma (between the chorion and the uterine wall, external to the chorion laeve), retroplacental hematoma (behind the placenta, external to the chorion frondosum), or both	anterior, posterior, fundal, or covering more than 1 site	––	––	yes
Özkaya et al. (2011)	prospective	study group: 43 patients with ultrasonographically detected subchorionic haemorrhage; control group: 45 age-matched group, without any abnormal ultrasonographic finding. (7–14 weeks, with vaginal bleeding)	––	SCH: crescentic fluid collection between the gestational sac and uterine wall	preterm labour (<37 weeks), spontaneous miscarriage (not defined), intrauterine growth restriction(IUGR)(birth weight smaller than 10 percentile of gestational age)	multiplication of three diameters divided by two	<32 ml, >32 ml	––	––	––	––	––	yes
Dongol et al. (2011)	prospective	70 women with vaginal bleeding in their first half of pregnancy	––	SCH (not defined)	spontaneous abortion (not defined)	––	<4 cm^2^, 4–20 cm^2^, >20 cm^2^	––	––	––	––	––	yes
Aoki et al. (2014)	retrospective	24 women with intermittent hemorrhage occurring throughout pregnancy (delivery at 22 weeks of gestation or later and presence of macroscopic retroplacental hematoma detected at delivery)	pregnancies with placenta previa and exclusion of cervical disease as the source of bleeding	persistent subchorionic hematoma and chronic abruption (not defined)	gestational age at delivery, acute abruption, SGA, neonatal chronic lung disease	––	––	––	––	––	in the first trimester or in the second to third trimester	persisting during pregnancy until delivery	yes

IUH, intrauterine hematoma; SCH, subchorionic hematoma; PTD, preterm delivery; SGA, small for gestational age.

–– indicates data not reported.

## Results

### The volume of IUH

**Figure 5:**
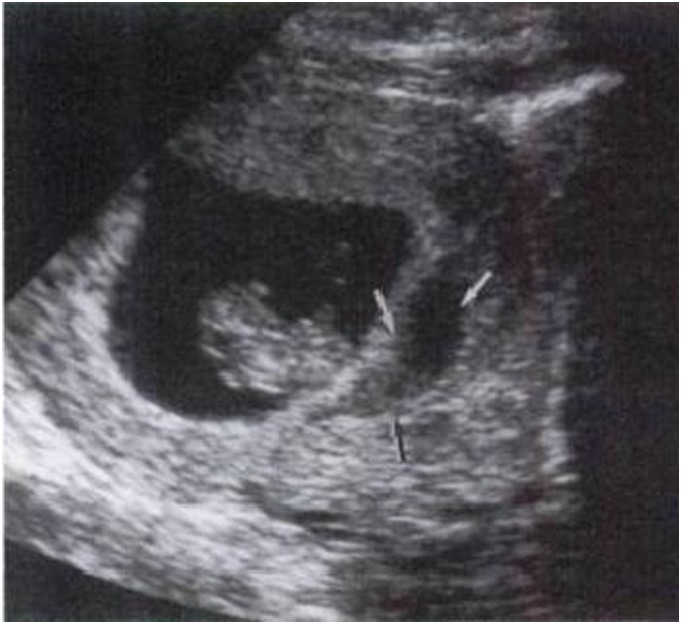
An 8-week fetus associated with a small (less than one-third of the chorionic sac circumference) subchorionic hematoma (arrows) [26].

**Figure 6:**
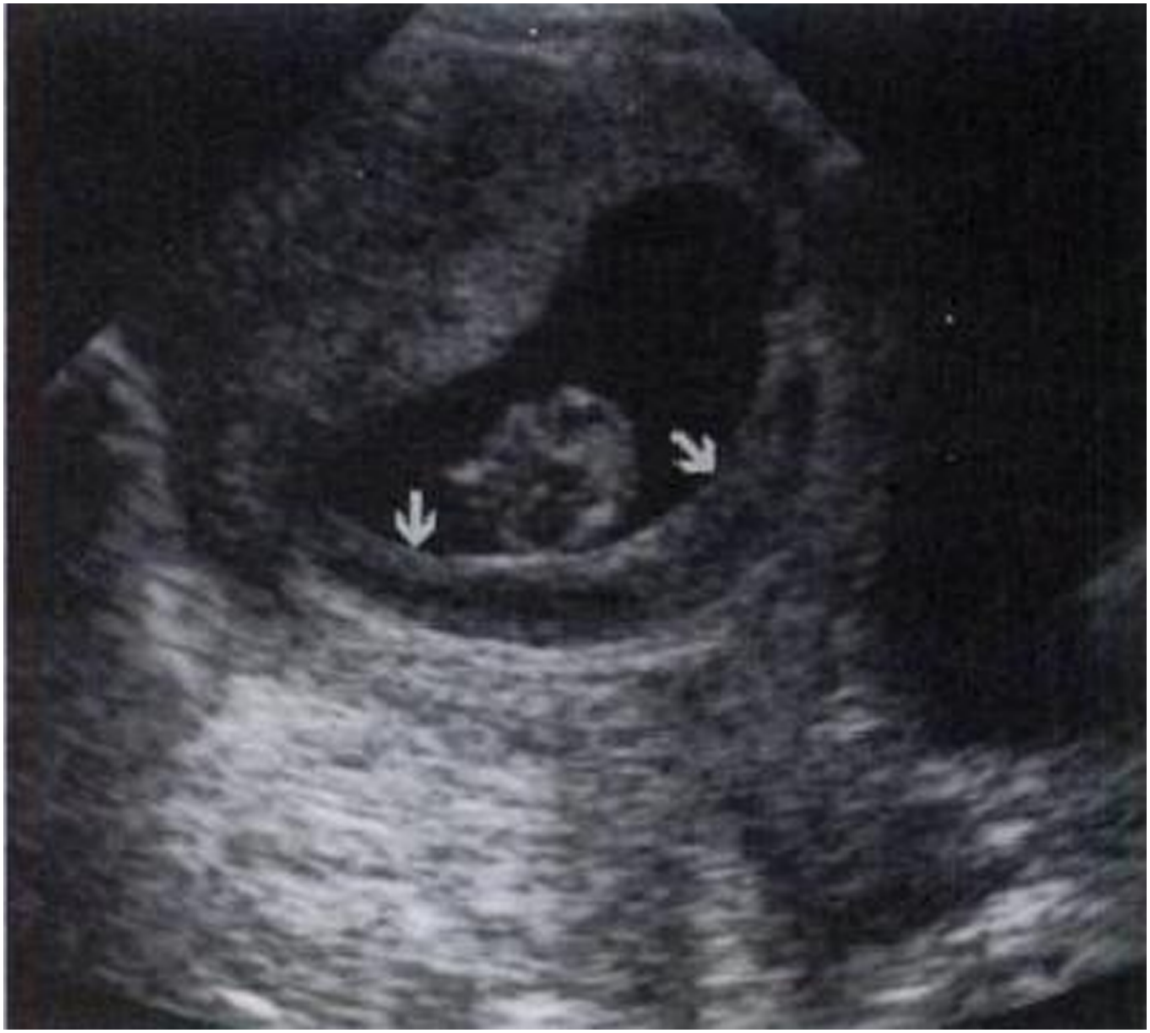
A 10-week fetal head adjacent to a moderate-size (one-third to one-half of the chorionic sac circumference) subchorionic hematorna (arrows) [26].

**Figure 7:**
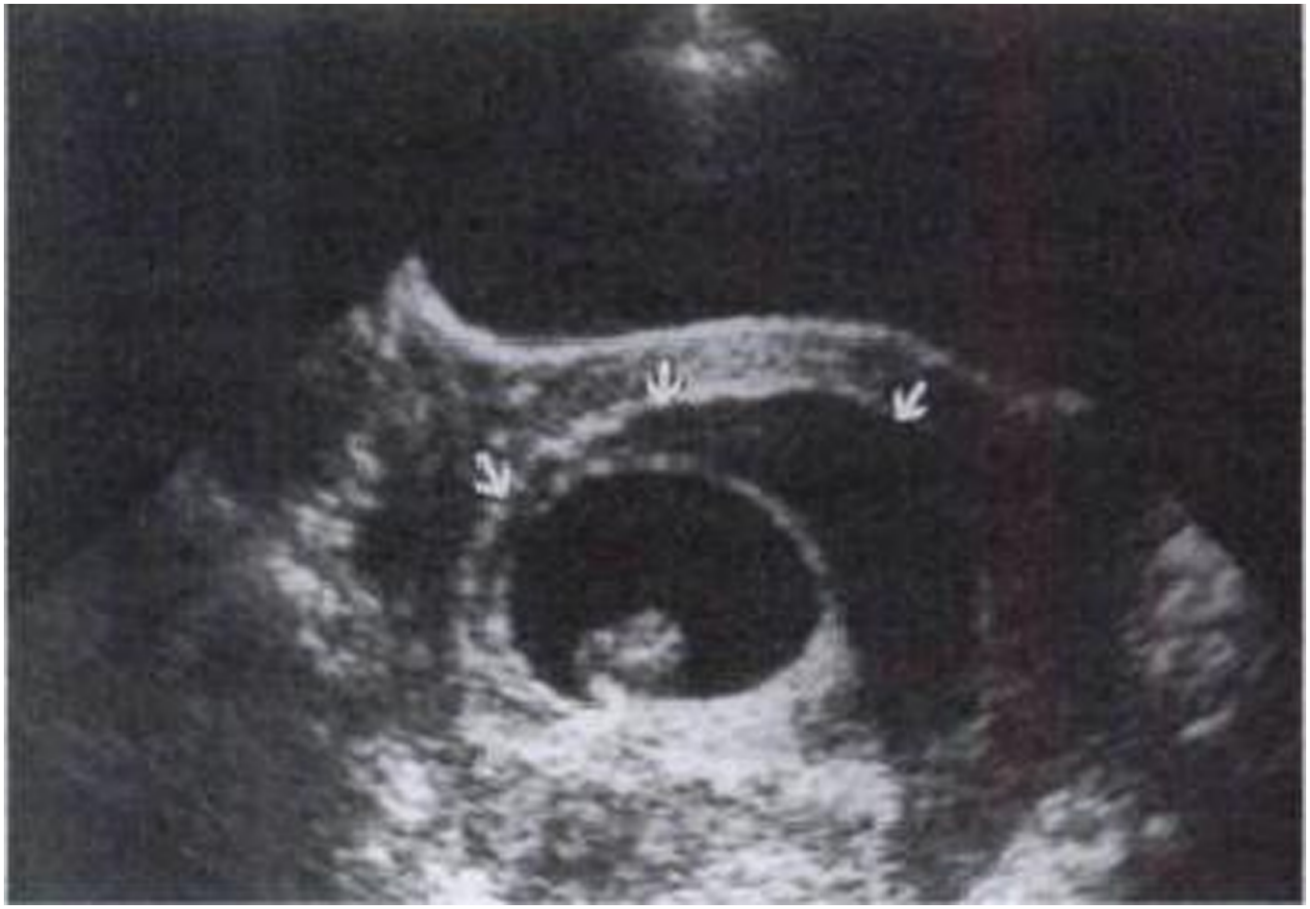
An 8-week fetus associated with a large (at least two-thirds of the chorionic sac circumference) subchorionic hematoma (arrows) [26].

Of the researches enrolled in our review, most reported the association between volume of a hematoma and pregnancy outcome. The calculation algorithm of a hematoma’s volume varied in different studies and that perhaps explained their different results. The earliest and easiest definition of absolute size was the multiplied product of longitudinal, transverse, and antero-posterior diameters [3], [14]. Since the outline of most hematomas was not regular rectangle, mean of its three diameters [15], [16], half of the product [9], [17]–[20], or the product multiplied by a constant [8], [21], [22], were used by later researchers. Some authors recorded the size directly by the largest diameter or area observed [23], [24]. Another was the relative size, which could be expressed in percentage. It was calculated as the absolute size of a hematoma relative to the gestational sac size [9]. The latter seemed to be more popular in recent reports [14], [21], [25]–[28]. No matter which method was used, the standard by which IUHs were classified as small, medium, and large in volume was also discrepant among studies (Figure 5, Figure 6 and Figure 7).

Even though the volume of a hematoma was described in many literatures, only a part of them made a formal statistical analysis to clarify whether or not there is a correlation between it and pregnancy outcome. Theoretically, the volume of hematoma should influence on the prognosis for pregnancy outcome. As Mantoni et al [3] reported, a hematoma of more than 50 ml occurring after 16 weeks of gestation would increase the risk of spontaneous abortion or PTD, while the smaller one (<35 ml) had a better prognosis. Results of Mandruzzato et al [29] (abortion, p = 0.022; PTD, p = 0.026) were similar to the former report, when using 15 ml to classify 62 cases into two groups. The poor outcomes of pregnancies with a SCH correlated well with both the absolute and relative hematoma size in the report by Abu-Yousef et al [9]. Both Sauerbrei et al [14] and Nyberg et al (p<0.01) [8] demonstrated the outcomes tended to be favorable when the absolute IUH volume was less than 60 ml or the relative volume was less than 40%, and considered the better prognosis of the absolute volume than the relative one. Nevertheless, the sample size was too small for a formal statistical analysis. In 1996, the association between spontaneous abortion and hematoma size was demonstrated by both univariate and multiple logistic regression analysis in a retrospective study. A hematoma of two-thirds or greater of the gestational sac circumstance was a good predictor of abortion when the analysis was adjusted for maternal age and gestational age at diagnosis (OR, 2.9; 95%CI, 1.2–6.8) [26]. Özkaya et al [20] used 32 ml as the grading standard and found that the volume of SCH was significantly larger in the miscarriage group than that of the normal delivery group (p = 0.003). Recently, a small prospective study also reported that the volume of a SCH showed a positive correlation with the incidence of abortion, especially when a hematoma larger than 4cm^2^ [30].

However, in most recent studies, authors failed to demonstrate this association. In the pregnancies with threatened abortion, the estimated volume of IUH did not significantly predict abortion or PTD [15], [17], [18], [23], [31]–[36]. The frequency of subchorionic bleeding, observed as fluid motion on Doppler ultrasound, was shown to have a positive correlation with the size of subchorionic fluid (p = 0.041).[28]. However, neither subchorionic fluid nor subchorionic bleeding were associated with embryonic death unless accompanied by clinically significant bleeding. Interestingly, only by abdominal ultrasound, not by vaginal or Color Doppler ultrasound, embryonic death occurred more often in the single pregnancies with a larger amount of subchorionic fluid (p<0.0001). Thus it can be presumed that the checking method for a hematoma is important to the prognosis. When comparing the impact of different size of SCH, no significant difference was observed with respect to the risk of abortion and neonatal death, but difference to that of stillbirth and total adverse outcomes (p<0.05) [25]. Rizzo et al [19] found the volume of retroplacental hematomas was similar between the groups with or without abortion (71.07±37.87 ml vs 60.55±30.62 ml, p>0.05). Recently, two retrospective studies also showed the hematoma volume did not correlate well with pregnancy outcomes (p>0.05) [16], [22]. As Nagy et al [21] reported, perhaps it was the presence or absence of a hematoma, not its size, could be used as a marker of the integrity of placentation. Both them and Sharma et al [24] supported the irrelative correlation whether in their prospective or retrospective studies (p>0.05).

Overall, the association between the volume of a hematoma and pregnancy outcome still remains controversial. However, the size of a hematoma may be a poor reflection of the accurate amount of hemorrhage because it depends upon the following factors: the ability of blood to escape through cervix [3]; the rate of intrauterine bleeding [25]; the time between the acute hemorrhage and the US scan [14]. So the influence of external bleeding on the pregnancy outcome in the pregnancies with IUH also needs further in-depth discussion. On that basis, Maso et al [22] postulated that both the presence and location of a hematoma which represented the impaired placentation, rather than its volume, were important for pregnancy outcome.

### Position or location of a hematoma

A number of studies described the position of a hematoma relative to the placental site, but details on the clinical significance were not involved [1], [3], [15], [17], [31], [34], [37]. The correlation between the degree of placental detachment and the pregnancy outcome was examined by recent studies. The prognosis was slightly better when only the placenta margin was separated, because the small fraction of placental surface that was involved did not materially affect the volume flow of fetal perfusion [14]. Later, Nyberg et al [8] also demonstrated fetal death correlated best with the estimated percentage of placental detachment.

SCHs often detach only the placental margin [3], [8], [14], [31], while retroplacental hematomas represent large placental detachment. SCH appears to result from tears of marginal veins, whereas abruption of placentae results from ruptures of spiral arteries [1], [38]. Therefore, the placental impairment seemed to be more serious in the retroplacental hematoma than in the SCH. None correlation between marginal placental elevation and pregnancy outcome probably indicated SCH was less dangerous [9]. Nyberg et al [8] demonstrated that the large retroplacental hemorrhages were more risky to the placental function than those small and/or subchorionic ones. Glavind et al [23] considered that a subplacental hematoma tended to be related to a higher, but not statistically significant risk of abortion than a subchorionic localization (p = 0.087). Comparing with SCH, retroplacental hematoma was correlated with a significantly increased risk for pregnancy complications, such as fetal mortality (p<0.01) [8], fetal distress, meconium-stained amniotic fluid, NICU admission (P<0.001), PTD (P = 0.001), preeclampsia (P = 0.007), and fetal growth restriction (P = 0.04) [21]. However, as an exception to SCH, chronic peripheral separation would result in release of hemoglobin, with its degradation products into the amniotic cavity, and eventually lead to diffuse chorioamniotic hemosiderosis (DCH), clinically known as chronic abruption [39], [40]. Pregnancy complicated by DCH is supposed to be closely associated with PTD and newborn respiratory diseases [41]–[43].

Either sustained uterine contraction induced by an IUH or the IUH itself influences the uterine blood flow and supply lines, further contributing to fetal hypoxia, which appears to be the primary cause of fetal death from placental abruptions [8], [29]. And the retroplacental hematoma itself may be associated with placental infarction due to small vessel disease of the maternal uterine spiral arteries [44]. Both of the two conditions are associated with an impaired placenta. Loss of placental function plus with sustained contractions will result in labor. Moreover, the chorionic villous hemorrhage preceding retroplacental hemorrhage reflects a disturbance of fetal vascular dynamics [45]. However, the majority of women with an IUH did not experience abortion or fetal mortality. A possible explanation was presented in an early report by Rizzo et al [19]. Fetal circulation was not influenced by retroplacental hematoma before 14 weeks. They postulated that placental damage caused by the hematoma was not severe enough to impair the maternal transferring of oxygen and nutrients to the fetus or the presence of low oxygen and nutritional requirements in the fetus of this gestational period could be satisfied even in the presence of the hematoma. A second possibility was the small fraction of retroplacental hematoma and chronic peripheral separation and large proportion of SCH documented in the published literatures. As Pedersen et al [17] thought the bleeding never tracked beneath the placenta. They supported that the placental function would not suffer, by the normal average birth weight (3,112 g) of the 20 babies born at term (average gestational age 275 days), in 23 pregnancies presenting with both clinical vaginal bleeding and a large hematoma (≥50 ml). It confirms that a hematoma alone is not an initiator of labor. In 2006, Leite et al [27] also proved that no significant difference was observed in the positions related to placenta between the favorable and non-favorable groups (p = 0.63). But they only compared the incidence of SCH plus retroplacental hematoma with that of presence of both two. In that study, which of the two hematomas was more influential to the prognosis for pregnancy outcome was not presented.

Another classification of IUH was by its relative position to the uterine wall. The earliest report demonstrated the hematoma’s location in the uterus was most often dependently on the posterior uterine wall, close to, or covering, the internal os. Moreover, the small hematomas (<5 ml) just inside the internal os did not affect the perinatal outcomes [3]. Nevertheless, if the placenta completely occupied the posterior wall, the hematoma would be located laterally and anteriorly [17]. The risk of fetal distress was significantly increased when the hematoma was located posteriorly (p = 0.04). But that of other pregnancy complications, such as meconium-stained amniotic fluid, preterm delivery, preeclampsia and fetal growth restriction, was not wherever the hematoma was located [21]. Comparing with the supracervical hematoma, a SCH in the corpus or fundus of uterus was associated with an increased risk of both spontaneous abortion and PTD (p = 0.03) [18]. Since the region of placental site was mostly in the corpus or fundus of uterus, Kurjak et al [18] then postulated that the placental function was possibly disrupted if the hematoma was located there. Recently, Leite et al [27] found that the prognosis for pregnancy outcomes was similar whether a hematoma covered one or more than one site (anterior, posterior, fundal) (p>0.9).

In a word, the position or location of a hematoma relative to the placenta or the uterine wall was important on the prognosis for pregnancy complications.

### Gestational age at diagnosis

Pedersen et al [33] found the size of a hematoma increased in proportion to the gestational age at the first ultrasonographic examination. If the size of a hematoma is related to pregnancy complications, a positive correlation should exist between the gestational age at diagnosis and outcomes. As mentioned above, only the large hematoma occurring after 16 weeks gestation had a deleterious effect on the outcomes [3]. The results of Nyberg et al [8] also indicated that a pregnancy with a hematoma debut after the 20^th^ week was followed more often by PTD than was that before (p<0.001). The similar finding was reported by Børlum et al [32] using the first trimester as the classification criterion. These data suggest that most early IUHs can be managed expectantly.

On the other hand, the view on the correlation was opposed by other researchers. Bennett et al [26] found a gestational age of 8 weeks or less was predictive of an increased percent of spontaneous abortion (OR, 2.6; 95% CI, 1.4–4.9). In 2005, Maso et al [22] reported the overall risk of adverse outcomes, especially for spontaneous abortion, was 2.4 times higher in the pregnancies with a hematoma observed before the 9^th^ week. Later researchers compared the median gestational ages at the first ultrasonographic examination and observed a significant difference between the favorable and non-favorable group (8.4^th^ week vs 7^th^ week, p = 0.0227) [27]. In the latest report by Aoki et al [46], the percentage of acute abruption tended to be higher, but not significantly (p = 0.129), in the group with hemorrhage occurring in the second to third trimester (chronic abruption) (66.7%) than that with the one starting from the first trimester (persistent SCH) (22.2%). However, the study proved significantly earlier gestational age at delivery (P = 0.017) and higher incidence of small for gestational age (SGA) infants and neonatal chronic lung disease in the persistent SCH group [46]. In general, the earlier gestational age a hematoma was diagnosed, the worse outcome the pregnancies tended to have. One reason for this may be, in the middle and late pregnancy, the gradually diminishing cervical barrier resulting in fast outflow and reduced intrauterine retention of blood when uterine hemorrhage occurs [37].

The association of the time at detection of hematoma between with pregnancy complications was inconclusive by aforementioned reports. However, some researchers drew the conclusion that there was no significant correlation between the gestational age and the pregnancy outcome (p>0.05) [9], [23]. The menstrual age was alike between the groups with or without abortion in the study of retroplacental hematoma (11.43±1.38 weeks vs 11.54±1.11 weeks, p>0.05) [19]. Ben-Haroush et al [16] compared the risk of abortion and PTD in the groups of 10–12 and 13–20 weeks’ gestation at diagnosis of SCH, without significant difference observed (p>0.05). In a recent study with only subchorionic collections included, both the median gestational age and trimester at detection were also similar in the PTD and term delivery groups (p>0.05) [24].

In short, the prognostic value of gestational age at diagnosis still remains controversial.

### Duration of a hematoma

Duration is another valuable characteristic of a hematoma in the prognosis for pregnancy complications. Though a few reports gave an incomplete description on the duration without deep analysis [34]. Some studies proved a none correlation between it with pregnancy outcomes (p>0.05) [35], others affirmed its clinical significance on the prognostic value. Mantoni et al [3] first reported the pregnancies with a persistent hematoma ended up with abortion or rupture of membranes. Herein, persistent hematomas were defined as more than 50 ml, whereas all hematomas smaller than 35 ml disappeared by the 20^th^ week. This phenomenon demonstrated the volume might affect the duration of a hematoma. The hematomas often decreased in size when the patients experienced intermittent asymptomatic vaginal bleeding as time went on. The report of Abu-Yousef et al [9] showed the risk for unfavorable outcomes was significantly decreased in the patients with a diminishing hematoma, while not in those with an increasing or no changing one. That indicated the duration of a hematoma did influence the pregnancy outcome. Goldstein et al [31] also found fetal death occurred in two pregnancies with persistent subchorionic fluid collection, but the collections in those continuing to term were reabsorbed over 4–7 weeks. Similar to that before, a study enrolling 22 patients with persistent SCH until delivery also reported the pregnancies ended in abortion or premature labor finally [1]. The incidence of persistent SCH until delivery was 0.46%, which was much lower than that of a hematoma detected in the first trimester (4–48%) [47]. Because almost half of the women with an IUH did not experience vaginal bleeding, Tower and Regan [5] then postulated that most hematomas would be resolved spontaneously. That suggested a persistent hematoma until delivery could be a severe type. Many researchers found the hematomas in most cases was a small fluid collection above the internal os of the cervix on the last ultrasonographic examination and totally disappeared before the 24^th^ or 25^th^ week [15], [33], [48] or by the end of the first trimester [5], but a definitive conclusion on the association between duration and pregnancy outcome was not provided in those studies.

In short, the majority of the published literatures suggested that a persistent hematoma would make a great contribution to adverse pregnancy outcomes.

### Influence of the presence of vaginal bleeding on pregnancy outcome

Most of the included patients in the previous studies were pregnancies with vaginal bleeding. In view of the above-mentioned close correlation between IUH and vaginal bleeding, we therefore concluded the influence of the simultaneous presence of vaginal bleeding on the pregnancy outcome. As mentioned in the volume part, neither subchorionic fluid nor subchorionic bleeding would increase the risk of embryonic death unless they were accompanied by clinical bleeding at the same time [28]. In the pregnancies with a SCH, those complicated by antepartum bleeding were more likely to deliver prematurely than those without bleeding (26.6% vs 7.0%, P = 0.009). So were those presenting with bleeding before SCH detection (25% vs 10.9%, P = 0.015) [24]. Jouppila [15] first reported a higher risk of spontaneous abortion, but a lower risk of PTD, occurred in the pregnancies with a total bleeding time of more than 14 days (25% vs 0 and 4.2% vs 22.2%). However, due to the limited sample size, they did not perform a formal statistical analysis. They further considered the duration of bleeding significantly influenced the time when the hematoma could be detectable, namely the gestational age at diagnosis (r = 0.68, p<0.01). The study by Mandruzzato et al [29] was similar to that before. They observed no abortions occurred in the group without bleeding and the risks for both abortion and PTD were higher in the patients with bleeding for longer than 14 days. Mantoni [48] stressed the presence of IUH plus bleeding for 3 days or more significantly increased the risk for the pregnancy complications. Then Abu-Yousef et al [9] also confirmed a direct correlation between the duration and severity of bleeding and pregnancy outcome. In brief, the presence of vaginal bleeding will aggravate the state of complications in the pregnancies with a hematoma. Moreover, the longer the bleeding lasts, the worse the prognosis is.

However, the results from other authors were different from those aforementioned reports. Stabile et al [36] reported none of the 22 threatened abortions with first trimester IUH experienced abortion. The pregnancies with an IUH were more likely to experience vaginal bleeding than those without, but the bleeding did not affect the pregnancy outcome [5], [21]. On the contrary to some previous studies, pregnancy outcomes were not significantly different when bleeding lasted more or less than 14 days (p>0.05) [16]. Recently, Leite et al [27] assigned 30 pregnancies with an IUH into 2 groups according to the pregnancy outcome and found the incidence of vaginal bleeding was similar in the groups with or without complications (P = 0.6792).

In 1996, Ball et al [25] did not study whether or not the presence of vaginal bleeding itself added the risk for pregnancy complications in the pregnancies with a SCH, but found the SCH conveyed an increased risk over and above that for vaginal bleeding alone. Recently, results of Dongol et al [30] indicated bleeding with or without hematomas was associated with poor pregnancy outcomes. The retention after vaginal bleeding was speculated to result in chronic inflammatory reaction and form a nidus for intrauterine infection leading to adverse outcome [49]. Therefore, the speculation, both hematoma and bleeding were related to a basic placental problem, which fundamentally induced adverse outcomes, and should not be considered independently, was a more accurate statement [25].

## Conclusions

In this systematic review, we described the associated symptoms of an IUH, including the volume, location, gestational age at diagnosis, duration and the simultaneous presence or absence of vaginal bleeding, which may play an important role in the prognosis for pregnancy outcome in the pregnancies with a hematoma. Since the presence of an IUH is common in the antenatal ultrasonographic examination, the subsequent high risk for adverse pregnancy outcomes becomes one of our main concerns in the antenatal care. Data from our literature review indicate that a retroplacental, posterior, or subchorionic in the fundus of uterus, and/or persistent IUH will bring worse prognosis in the ongoing pregnancies. It seems that the prognosis of the volume and gestational age at diagnosis still remained hugely controversial up to now. Moreover, the value of the simultaneous presence of vaginal bleeding deserves more attention. One of the reasons for arguments may be due to the varied definitions in the published reports. Therefore, it is necessary to confirm the significance of the volume and gestational age at detection of a hematoma using a uniform criterion in the future.

To our knowledge, recent studies often assessed and compared the pregnancy outcomes between the groups with or without an IUH. But they ignored the impact of its concrete characteristics, such as the size, position, gestational age at diagnosis, duration or the simultaneous presence or absence of vaginal bleeding [2], [50], [51]. Even though many studies compared the symptoms in relation to the pregnancy complications, most of them were limited or small uncontrolled series, including without a clear definition of hematomas’ type and poor classification criteria as mentioned. Moreover, the correlation assessments of pregnancy outcome from previous studies were often incomplete, too. Hence, further prospective large cohort studies are needed to take all the detail symptoms of a hematoma into consideration when we evaluate its clinical significance in the prognosis of pregnancy.

## Supporting Information

Checklist S1 **PRISMA checklist.**
(DOC)
